# BMP Signaling Regulates Bone Morphogenesis in Zebrafish through Promoting Osteoblast Function as Assessed by Their Nitric Oxide Production

**DOI:** 10.3390/molecules20057586

**Published:** 2015-04-24

**Authors:** Thomas Windhausen, Steeve Squifflet, Jörg Renn, Marc Muller

**Affiliations:** Laboratory for Organogenesis and Regeneration, Université de Liège, GIGA-R B34, Sart Tilman, 4000 Liège, Belgium; E-Mails: t.windhausen@doct.ulg.ac.be (T.W.); steeve.squifflet@gmail.com (S.S.); jrenn@ulg.ac.be (J.R.)

**Keywords:** zebrafish, BMP, cartilage, bone, nitric oxide

## Abstract

Bone morphogenetic proteins (BMPs) control many developmental and physiological processes, including skeleton formation and homeostasis. Previous studies in zebrafish revealed the crucial importance of proper BMP signaling before 48 h post-fertilization (hpf) for cartilage formation in the skull. Here, we focus on the involvement of the BMP pathway between 48 and 96 hpf in bone formation after 96 hpf. Using BMP inhibitors and the expression of a dominant-negative BMP receptor, we analyze whether the loss of BMP signaling affects osteoblastogenesis, osteoblast function and bone mineralization. To this end, we used the transgenic zebrafish line *Tg(osterix:mCherry)*, detection of nitric oxide (NO) production, and alizarin red staining, respectively. We observed that inhibition of BMP signaling between 48 and 72 hpf led to a reduction of NO production and bone mineralization. Osteoblast maturation and chondrogenesis, on the other hand, seemed unchanged. Osteoblast function and bone formation were less affected when BMP signaling was inhibited between 72 and 96 hpf. These results suggest that for the onset of bone formation, proper BMP signaling between 48 and 72 hpf is crucial to ensure osteoblast function and ossification. Furthermore, detection of NO in developing zebrafish larvae appears as an early indicator of bone calcification activity.

## 1. Introduction

In zebrafish, major parts of the craniofacial skeleton derive from cranial neural crest cells (cNCCs) that previously migrated from the dorsal edge of the neural tube to differentiate into chondrocytes within the ventrally located pharyngeal arches [1]. Each pharyngeal arch is formed by a mesoderm-derived core embedded in the neural crest-derived cartilage precursor cells and is surrounded medially by endoderm and laterally by ectoderm. For formation of perichondral bone elements, the cartilage structures are populated with osteoblastic cells that will finally secrete the bone matrix. Depending on hedgehog signaling, the chondrocytes themselves or peripheral cells are recruited to that end [2]. Other (dermal) bone elements are directly formed by osteoblasts derived from mesenchymal cells, without a previous cartilage matrix. In mammals, one of the major genes involved in osteoblast differentiation is Runx2, similar to its zebrafish ortholog runx2b [3]. Further marker genes expressed in these bone-forming cells code for the transcription factor Osterix (Osx) [4] and bone extracellular matrix (ECM) proteins osteocalcin (Osc2) [5], collagen10a1 (Col10a1a) and collagen1a1a (Col1a1a) [6,7]. Finally, correct calcification of the bone ECM depends on transcellular epithelial calcium uptake through the calcium channel Trpv5/6 [8] and the precise control of phosphate/pyrophosphate homeostasis by the osteoblast-specific Entpd5 diphosphohydrolase [9] or the widely expressed phosphodiesterase Enpp1 [10].

In vertebrates, the bone morphogenetic protein (BMP) signaling pathway is known to play an essential role in many early developmental processes such as gastrulation or neurulation [11], but also in skeletogenesis [12]. BMP ligands bind to their transmembrane receptor complex, consisting of a type I and a type II receptor, to induce phosphorylation of the type I receptor. The activated receptor (Alk1, 2, 3, or 6) then phosphorylates Smad1, 5, and/or 8 which in turn associate with their common partner, Smad4 to migrate into the nucleus and regulate target genes [13]. Craniofacial defects were reported in conditional knock-out mice lacking BMP type I receptor Alk2 [14] or Smad4 [15] in cNCC, or in transgenic mice expressing the antagonistic Smad7 in cNCC cells [16]. In mammals, BMP2 is an important positive regulator for osteoblast differentiation by stimulating Osterix and Runx2 expression via Dlx5 [17,18]. Similar to the human bone disorder *osteogenesis imperfecta*, zebrafish harboring a mutation in the *bmp1a* gene display a higher mineral content of mature bone probably due to a negative effect on the ability to generate mature collagen fibrils [19]. In zebrafish, several members of the BMP ligand family, such as Bmp2a, Bmp2b Bmp4, Bmp5, Bmp7 were shown to be secreted in the pharyngeal region [12,20,21] and their importance for head cartilage development was shown [22]. Recently, BMPs were shown to promote ventral fates of the craniofacial skeleton in zebrafish before 24 hpf [23]. At later stages (30–36 hpf), the precise control of the expression of the *fsta* gene, encoding a BMP antagonist, in pharyngeal endoderm was shown to be required for the optimal amount of BMP signaling, required for proper chondrocyte differentiation and pharyngeal cartilage formation. Thus, the role of BMP signaling in skeleton formation has been extensively studied during the first two days of development, however little is known about its role at later stages, beyond 48 hpf.

Here, we show that BMP signaling is required between 48 and 72 hpf, and to a lesser extent between 72 and 96 hpf, for bone mineralization in the head skeleton. Inhibition of BMP signaling mainly affects osteoblast function, as assessed by monitoring their nitric oxide (NO) production, without affecting their proliferation or terminal differentiation.

## 2. Results

### 2.1. Inhibition of BMP Signaling Starting at 2 or 3 dpf Stages Affects Bone Mineralization

To assess the role of BMP signaling in head skeleton formation at later stages of development without affecting earlier processes, we investigated the effects of dorsomorphin, an inhibitor of ALK2, BMPR-IA and BMPR-IB signaling and of BMP-induced Smad1/5/8 phosphorylation [24] at different stages beyond 48 hpf.

**Figure 1:**
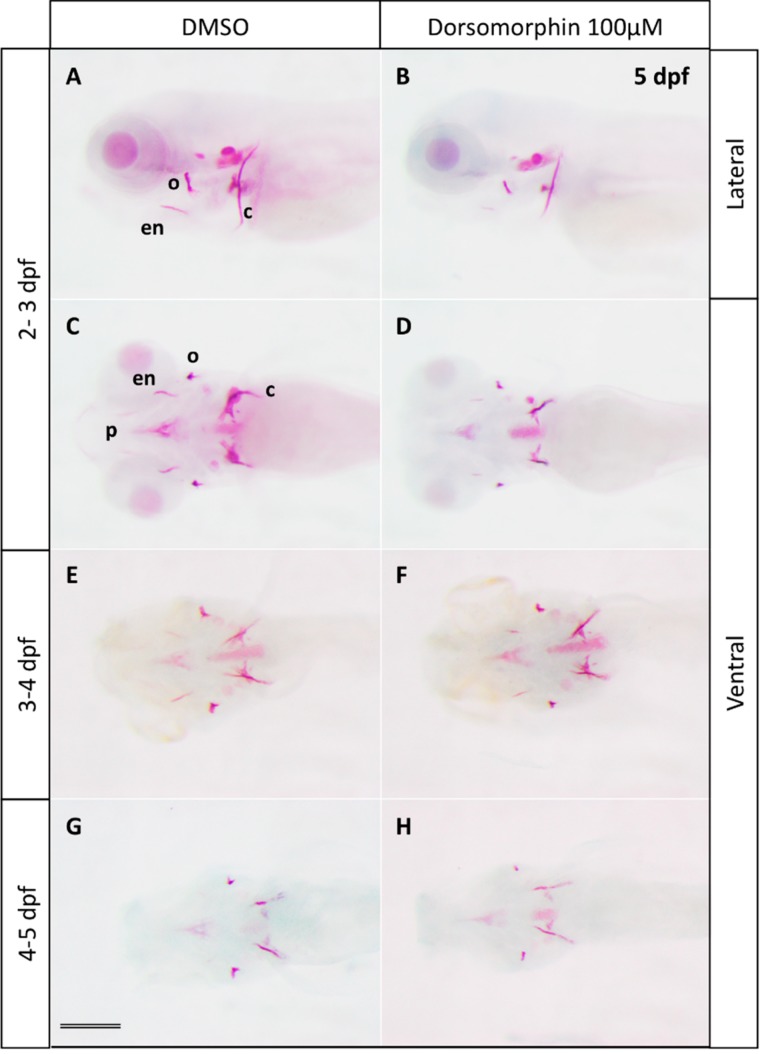
Effects of the BMP inhibitor dorsomorphin on bone mineralization. Alizarin red staining of 5 dpf larvae treated at 2, 3 or 4 dpf during 24 h with 100 µM dorsomorphin. Control embryos were treated with DMSO. Embryos treated at 2 dpf (**B** lateral view and **D** ventral view) show severe reduction of all mineralized bone pieces compared to the controls (**A** lateral view and **C** ventral view). The treatments starting at 3 dpf (**F** in ventral view) and 4 dpf (**H** in ventral view) lead to a reduction of bone mineralization, but to a lesser extent than at 2 dpf compared to the controls embryos (respectively **E** and **G** in ventral view). c: cleithrum; en: entopterygoid; o: operculum; p: parasphenoid. Scale bar: 200 µM.

Treatment of embryos with 100 μM dorsomorphin was performed for 3 different periods: between 48 and 72 hpf (2–3 dpf), between 72 and 96 hpf (3–4 dpf) and between 96 and 144 hpf (4–5 dpf), and cranial ossification was analyzed by Alizarin Red staining at 5 dpf (Figure 1 and Figure S1). Treatment during 2–3 dpf lead to a clear reduction in calcification of all the bone elements in more than 95% of the larvae, treatment during 3–4 dpf caused minor defects mainly in the branchiostegal rays 1, while treatment during 4–5 dpf caused no detectable defects relative to the corresponding controls (Figure 1 and Figure S1).

**Figure 2:**
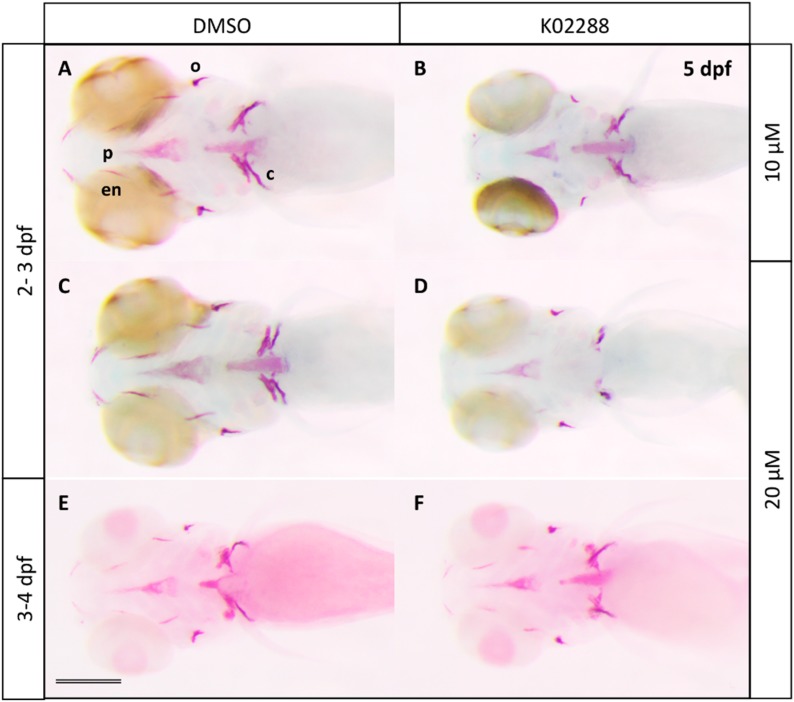
Effects of the specific BMP inhibitor K02288 on bone mineralization. Alizarine red staining of 5 dpf larvae previously treated at 2 (**B**,**D**) or 3 dpf (**F**) during 24 h with K02288 10 (B) and 20 µM (D,F). Control embryos (**A**,**C**,**E**) were treated with DMSO. c: cleithrum; en: entopterygoid; o: operculum; p: parasphenoid. Scale bar: 200 µM.

Although dorsomorphin was the first inhibitor of BMP signaling to be discovered [24], it was later shown to also inhibit vEGFR2 and AMPK signaling [25,26,27]. New generation BMP inhibitors were thus developed, among which the compound K02288 that presents a high specificity for BMP type I receptors, ALK1, 2, 3, and 6 [28] and was shown to induce dorsalization in early embryos at 8–10 µM concentrations. When we tested K02288 treatment on developing zebrafish larvae at two different concentrations, we observed a clear decrease in bone calcification at both 10 or 20 µM upon exposure from 2–3 dpf, while only a weak effect was observed following a 3–4 dpf treatment at 10 (not shown) or 20 µM K02288 (Figure 2 and Figure S2). Higher concentrations (40 µM) resulted in generation of morphological defects, such as cardiac edema, and were thus not considered.

**Figure 3:**
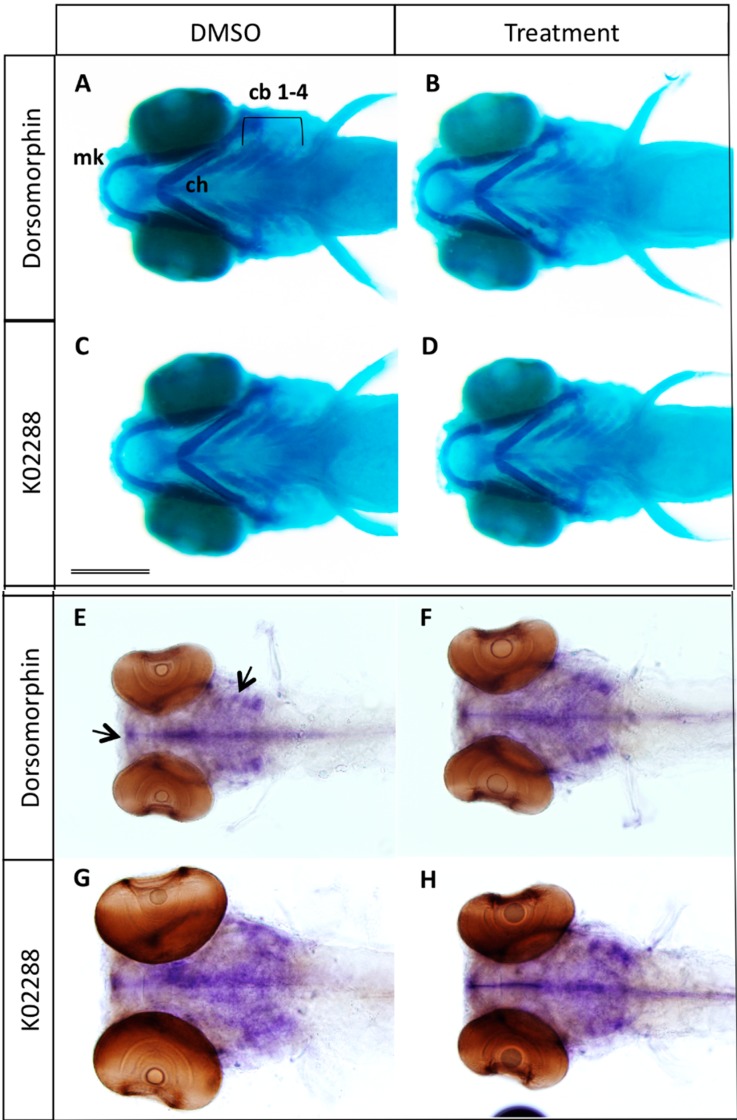
(**A**–**D**) Effects of BMP inhibitor treatment at 2–3 dpf on cartilage formation. Alcian blue staining of 5 dpf larvae previously treated at 2 dpf during 24 h with 100 µM dorsomorphin (B) or 20 µM K02288 (D). Control embryos (A,C) were treated with DMSO; (**E**–**H**) *In situ* hybridization using the *sox9a* probe. No difference in cartilage formation was observed between control (E,G) and treated (F,H). cb 1–4: ceratobranchial ray 1 to 4; ch: ceratohyal; mk: Meckel’s cartilage. Scale bar: 200 µM.

To evaluate the specificity of the observed effects for bone formation, we also checked formation of the cartilage skeleton that serves as matrix for most of the cranial bone formation. Treatment with dorsomorphin or K02288 was performed from 2–3 dpf and cartilage extracellular matrix was stained with Alcian Blue at 5 dpf (Figure 3 and Figure S3). No defect was observed in the cranial cartilage formation, suggesting that BMP signaling at later stages mainly acts on bone formation (Figure 3 and Figure S3). Whole mount *in situ* hybridization on 5 dpf revealed a similar expression pattern for the cartilage-specific *sox9a* gene in control and inhibitor treated larvae (Figure 3E–H, arrows). In living embryos, cartilage formation can additionally be assessed by nitric oxide labeling [29] (see below).

### 2.2. Type I BMP Receptors Are Required for Osteoblast Formation and Function

**Figure 4:**
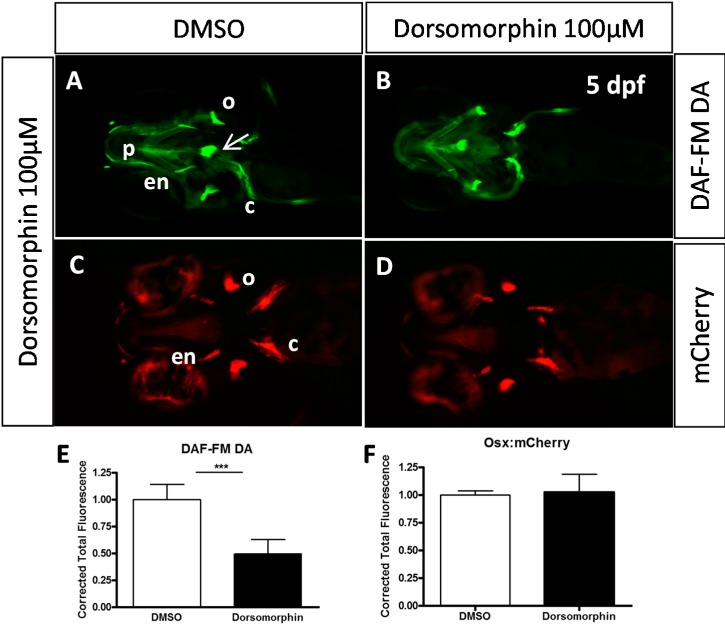

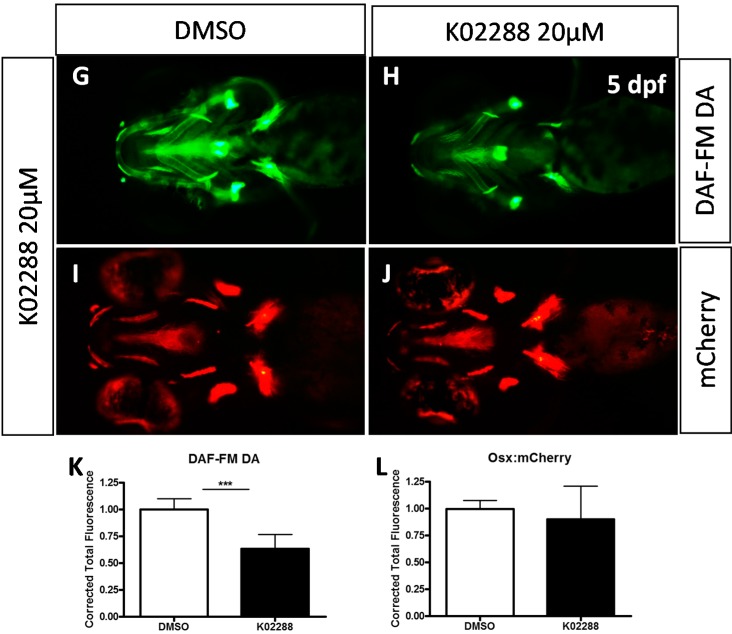
Effects of dorsomorphin (**A**–**F**) and K02288 (**G**–**L**) on NO production and osterix expression. (A,B,G,H) Nitric oxide labelling by DAF-FM DA of 5 dpf *Tg (osx:mcherry)* larvae previously treated during 24 h with 100 µM dorsomorphin (B) or 20 µM K02288 (H) at 2 dpf. (C,D,I,J) mCherry red fluorescence of 5 dpf *Tg (osx:mcherry)* larvae previously treated during 24 h with 100 µM dorsomorphin (D) or 20 µM K02288 (H) at 2 dpf. Control embryos (A,C,G,I) were treated with DMSO. c: cleithrum; en: entopterygoid; o: operculum; p: parasphenoid. Scale bar: 200 µM. (E,F,K,L) Statistical analysis of replicate experiments investigating the effect of dorsomorphin (E, F) or K02288 (K,L) on nitric oxide (E,K) or mCherry (F, L) detection. *** indicates *p*-value < 0.005.

Secretion of the bone extracellular matrix and subsequent mineralization requires the presence and activity of mature osteoblasts. We therefore decided to investigate whether osteoblast differentiation and/or function would be affected by inhibition of BMP signaling. One of the most prominent marker genes for osteoblast differentiation, both in mammals and in teleosts, is the Osterix gene *osx* [4,30,31]. The transgenic zebrafish line *Tg(osterix:mCherry)* contains the coding sequence for the red fluorescent protein mCherry under the control of the medaka *osterix* promoter, thus allowing detection of osteoblasts in living larvae. When we treated these transgenic larvae at 2 dpf with dorsomorphin or K202288, we observed a similar pattern of expression both in treated and control larvae at 5 dpf (Figure 4C,D,F,I,J,L), suggesting that osteoblast proliferation and terminal differentiation were not affected by this treatment. Only the most anterior structures, the dentary and maxillary were less intensely fluorescent in the treated larvae. Since the number of differentiated osteoblasts in the cranial skeleton did not seem to be generally affected by BMP inhibition, we decided to probe the function of these osteoblasts. In addition to producing and secreting ECM proteins such as collagens, osteocalcin or osteopontin, one striking feature of these cells in the developing embryo is their production of nitric oxide (NO).

Indeed, detection of NO in 5 dpf larvae using the green fluorescent label DAF-FM DA reveals strong staining of all the cranial bone elements and weak staining of cartilage structures; only the developing heart is stained to a similar extent (Figure 4A, arrow) [29]. NO detection in larvae treated at 2 dpf for 24 h with dorsomorphine or K02288 revealed a decrease of NO production in all bone elements (Figure 4A,B,E,G,H,K). The weak staining of cartilage structures was not affected.

### 2.3. Expression of a Dominant-Negative BMP Receptor Affects Bone Formation

**Figure 5:**
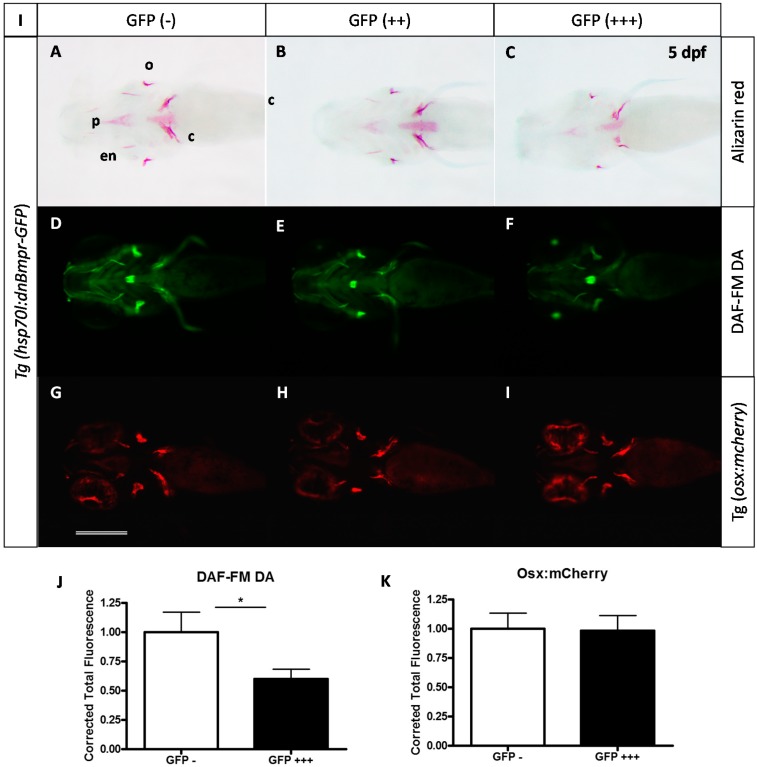
Effects caused by expression of the dominant negative Bmpr on bone formation. (**A**–**C**) Alizarin red staining of 5 dpf larvae after heat-shock treatment at 2 or 3 dpf. The extent of GFP expression 2 h after treatment indicates the transgenic status of the larva; GFP(−) being non-transgenic, GFP(++) transgenic, GFP(+++) being homozygous transgenic or larvae highly expressing the transgene (*dnBmpr-GFP)*; (**D**–**F**) DAF-FM DA staining for nitric oxide detection in 5 dpf larvae (note that GFP fluorescence has disappeared at this time); (**G**–**I**) Red fluorescence allowing detection of mCherry-expressing osteoblasts c: cleithrum; en: entopterygoid; o: operculum; p: parasphenoid. Scale bar: 200 µM. (**J**,**K**) Statistical analysis of replicate experiments investigating the effect of dnBmpr expression on nitric oxide (J) or mCherry (K) detection. * indicates *p*-value < 0.05.

To confirm this requirement for BMP signaling at late stages, we also used the transgenic line *Tg(hsp70l:dnBmpr-GFP)w30* harbouring a heat-shock inducible gene for a fusion protein between a dominant negative BMP receptor and the GFP. We performed a heat shock at 37 °C during 30 min at 2 dpf and compared the head skeleton at 5 dpf of the transgenic larvae to that of their non-transgenic siblings (Figure 5A–C), as determined by their expression of the GFP fusion protein. While all control larvae formed a perfectly normal skeleton, indicating that the heat shock itself had no effect, the transgenic larvae (positive for GFP expression) producing the active dominant negative BMP receptor presented a clear decrease of bone calcification. This effect was strongest in those individuals presenting the highest GFP expression. When heat shock on *Tg(hsp70l:dnBmpr-GFP)w30* was performed after 48 hpf, 123 of 129 larvae presented normal cartilage (data not shown). These results clearly confirm that BMP signaling is required for bone formation between 48 hpf and 72 hpf, without affecting cartilage formation.

We further investigated the extent of osteoblast differentiation by crossing parents of the *Tg(hsp70l:dnBmpr-GFP)w30* with those of the *Tg(osterix:mCherry)* line and by inducing dnBmpr expression in the larvae by heat shock at 48 hpf. We observed no significant difference in mCherry expression in the double transgenic larvae (Figure 5G–I,K), thus confirming that inhibition of BMP signaling after 48 hpf does not affect osteoblast formation. In contrast, NO detection in the same animals revealed a significant decrease of osteoblast activity at 5 dpf in the transgenic animals after heat shock at 2 dpf (note that the fluorescence of the dnBmpr-GFP fusion protein observed at 2–3 dpf is nearly absent at 5 dpf, except in the lens) (Figure 5D–F,J).

## 3. Discussion

The role of BMP/TGFβ ligands and/or antagonists in early embryonic development has been extensively documented in fundamental processes such as dorso-ventral patterning or establishment of left-right asymmetry [32,33,34]. Accordingly, many studies on the function and modulation of BMP signaling have concentrated on early stages of development, also concerning skeletal development [12,35]. Recent studies showed the involvement of BMP signals in cNCC migration and induction of pre-chondrocyte determination both in mouse [36] and in zebrafish around 12 hpf [37]. Later, at post-migratory cNCC stages in zebrafish (20–24 hpf), local BMP sources control the dorso-ventral patterning of the cranial skeleton [23,38]. Finally, BMP signaling was shown to be required around 30–36 hpf for induction of the runx2b gene in the differentiating chondrocytes, while inhibition of the BMP pathway at 48 hpf did not affect chondrocranium formation [39].

Based on these results, we investigated the role of BMP signaling at these later stages on cranial bone formation, osteoblast differentiation, and bone mineralization. Using two different BMP inhibitors, dorsomorphin [24] and K02288 [28], we show that BMP signaling is required between 48 and 72 hpf, and to a lesser extent between 72 and 96 hpf, for cranial bone ossification as revealed by alizarin red staining of the bone matrix. This result was confirmed by inducing the expression of a dominant-negative Bmp receptor [40] at 48 or 72 hpf. Note that we observed expression of the dnBmpr-GFP fusion protein through its green fluorescence already 2 h after the heat shock, indicating that the delay caused by transcription-translation is minimal. Taken together, these observations define the period between 48–72 hpf as the most crucial for BMP induction of ossification in the head skeleton. To further characterize the function of BMP signaling during these later stages, we investigated the differentiation and function of the osteoblasts upon pathway inhibition. Both the treatment with chemical inhibitors and expression of a dominant negative receptor only marginally affected the number of Osterix-expressing osteoblasts in the head of 5 dpf larvae, indicating that both proliferation and differentiation of these cells does not significantly depend on BMP signaling during this period.

Members of the TGFβ family have also been shown to be involved in zebrafish early cartilage development, such as Tgfβ2 [41] and Tgfβ3 [42]. Although these studies were performed using antisense morpholino injection and thus blocked gene expression from very early on, these factors may also affect skeletal development at later stages as studied here. The K02288 inhibitor is more than 100-fold more potent against BMP receptors ALK1 and ALK2 as compared to TGFß receptors ALK4 and ALK5 [28], while we found no evidence that the dnBmpr could interact with ALK4 or ALK5, therefore we think that the results presented here are due to inhibition of BMP signaling. Future studies may use specific TGFβ inhibitors to investigate the function of these factors in late skeletogenesis.

Nitric oxide (NO) has long been associated to bone development and pathologies [43,44]. Pharmacological inhibition of nitrogen oxide synthases (NOS) enzymes in rats leads to a decreased bone mass [45] and impairment of tibial growth [46], while mice deficient in endothelial NOS (eNOS) present reduced bone volume and severe defects in osteoblast maturation and activity [47,48]. NO production by osteoblasts has been associated with pathological conditions, such as inflammatory bone loss through activation of the cGMP/protein kinase G (PKG) pathway inducing Runx2 expression and production of matrix metalloproteinase MMP13 [44,49,50]. Bone cells are also known to react to mechanical or shear stress by producing nitric oxide [51,52,53], while NO action in shear stressed human and mouse osteoblasts was shown to result in activation of PKG, the intracellular protein kinases Src and Akt [54] finally inducing nuclear translocation of β-catenin [55]. Similarly, early osteogenic differentiation of murine embryonic stem cells as well as their mineralization was shown to be enhanced by NO, acting on ß-catenin and involving the canonical Wnt pathway [56].

In zebrafish, inhibition of BMP signaling between 48–72 hpf did not significantly affect osteoblast differentiation. In contrast, production of nitric oxide was clearly decreased in the treated animals. This observation, together with the observed decrease in cranial bone mineralization, leads to the conclusion that NO production in developing cranial osteoblasts in zebrafish is closely associated to bone calcification.

## 4. Experimental Section

### 4.1. Fish and Embryo Maintenance

Zebrafish (*Danio rerio*) were reared in a recirculating system from Techniplast (Buguggiate, Italy) at a maximal density of 7 fish/L. The water characteristics were as follows: pH = 7.4, conductivity = 50 mS/m temperature = 28 °C. The light cycle was controlled (14 h light, 10 h dark). Fish were fed twice daily with dry powder (ZM fish food^®^, Zebrafish Management Ltd, Winchester, UK) with size adapted to their age, and once daily with fresh nauplii from *Artemia salina* (ZM fish food^®^). Larvae aged less than 14 days were also fed twice daily with a live paramecia culture. Wild type embryos from the AB strain were used and staged according to Kimmel [57]. The transgenic line *Tg(hsp70l:dnBmpr-GFP)* [40] was obtained from the Zebrafish International Research Center (ZIRC, Eugene, OR, USA). *Tg(osterix:mCherry)* transgenic zebrafish have been generated as described earlier [4,29,30].

The day before breeding, two males and two females were placed in breeding tanks out of the recirculating system, with an internal divider to prevent unwanted mating. On the day of breeding, fish were placed in fresh aquarium water and the divider was removed to allow mating. Eggs were raised in E3 (5 mM Na Cl, 0.17 mM KCl, 0.33 mM CaCl_2_, 0.33 mM MgSO4, 0.00001% methylene blue).

### 4.2. Alcian Blue and Alizarin Red Staining

Cartilage and bone staining using alcian blue (CAS No: 33864-99-2) and alizarin red S (CAS No: 130-22-3, Sigma-Aldrich, Diegem, Belgium), respectively, on fixed specimen were performed as previously described [58].

### 4.3. Inhibition of Bmp Signaling

Transgenic embryos hsp70l:dnBmpr-GFP [40] were heat shocked at desired stages by placing them into a water bath for 30 min at 37 °C and afterward placed back at 28 °C. Two hours after the heat shock, the embryos were screened for GFP fluorescence. Embryos not expressing GFP were used as non-transgenic controls.

Inhibition of Bmp signaling 10 mM stock solution of dorsomorphin (CAS No: 1219168-18-9) (Sigma-Aldrich), K02288 (CAS No: 1431985-92-0) (Tocris Bioscience^®^, Lille, France) was diluted in DMSO (Merck Chemicals, Overijse, Belgium). Embryos at desired stage were placed into 6-well plates with the inhibitor diluted in E3 rearing medium at the desired concentration during a specific time period. DMSO alone was used as control (1% for dorsomorphin 100 µM and 0.1% and 0.2% for K02288 10 µM and 20 µM respectively). Embryos were then rinsed several times with E3, raised in E3 and finally fixed at the desired stage.

All experiments were performed on at least 20 individuals and repeated at least three times. Images shown in the figures are representative for more than 95% of the larvae observed under the respective conditions. Staining intensity was evaluated based on overall intensity, and focusing on the less intensely stained elements.

### 4.4. Living Nitric Oxide Labeling

For labeling of nitric oxide (NO), DAF-FM DA stock (CAS No: 254109-22-3) (Alexis corporation, Lausen, Switzerland; 5 mM in DMSO) has been diluted 1:1000 in E3 zebrafish medium and larvae were incubated over night at 28 °C in the dark and rinsed several times before imaging as previously described [29,59].

### 4.5. Whole-Mount in Situ Hybridization (WISH)

Whole mount *in situ* hybridization was performed as previously described [60]. The antisense RNA probe for *sox9a* [61] was synthesized by transcription of cDNA clones with T7, T3 or SP6 RNA polymerase using digoxigenin labeling mix (Roche Diagnostics, Meylan, France). Treated and non-treated larvae were fixed at 5 dpf in PFA 4%. *In situ* labeling was observed using an Olympus SZX10 stereomicroscope (Olympus, Berchem, Belgium) coupled with an Olympus XC50 camera.

### 4.6. Image Aquisition

All images were taken on a SZX10 stereomicroscope driven by the CELL B software (Olympus). Fluorescent images for nitric oxide labeling and transgenic mCherry expression were obtained using GFPA (Exc.: 460–490, Em.: 510–550) and RFP (Exc.: 540–580, Em.: 610) filters, respectively. For live imaging, embryos were anaesthetized with tricaine (Sigma-Aldrich) until they showed sufficiently low movement.

Quantification of fluorescence was performed using the ImageJ software [62] by first isolating the red or green channel, followed by integration of the pixel intensity on the resulting grey scale image. For nitric oxide labeling, the integrated density of the heart was subtracted, as this staining does not correspond to osteoblasts. The obtained numbers were normalized by dividing all values by the mean value obtained for the control larvae, and statistical significance of the observed differences was evaluated using a two-tailed t-test.

## 5. Conclusions

In conclusion, we show here that BMP signaling is of major importance for cranial bone formation in zebrafish between 48–72 hpf, with only minor effects on osteoblast proliferation and differentiation, but mainly by affecting bone mineralization through induction of osteoblast activity as visualized by their production of nitric oxide. Further, it appears that detection of nitric oxide in developing zebrafish larvae may be an early indicator of bone calcification activity.

## Supplementary Materials

Supplementary materials can be accessed at: http://www.mdpi.com/1420-3049/20/05/7586/s1.
