# Supplementary material for: BMP Signaling Regulates Bone Morphogenesis in Zebrafish through Promoting Osteoblast Function as Assessed by Their Nitric Oxide Production
Source: Molecules. 2015 Apr 24;20(5):7586–601. doi: 10.3390/molecules20057586 (PMC6272212; doi:10.3390/molecules20057586)
Supplement: Supplementary file 1 [file molecules-20-07586-s001.pdf]

Supplementary Materials

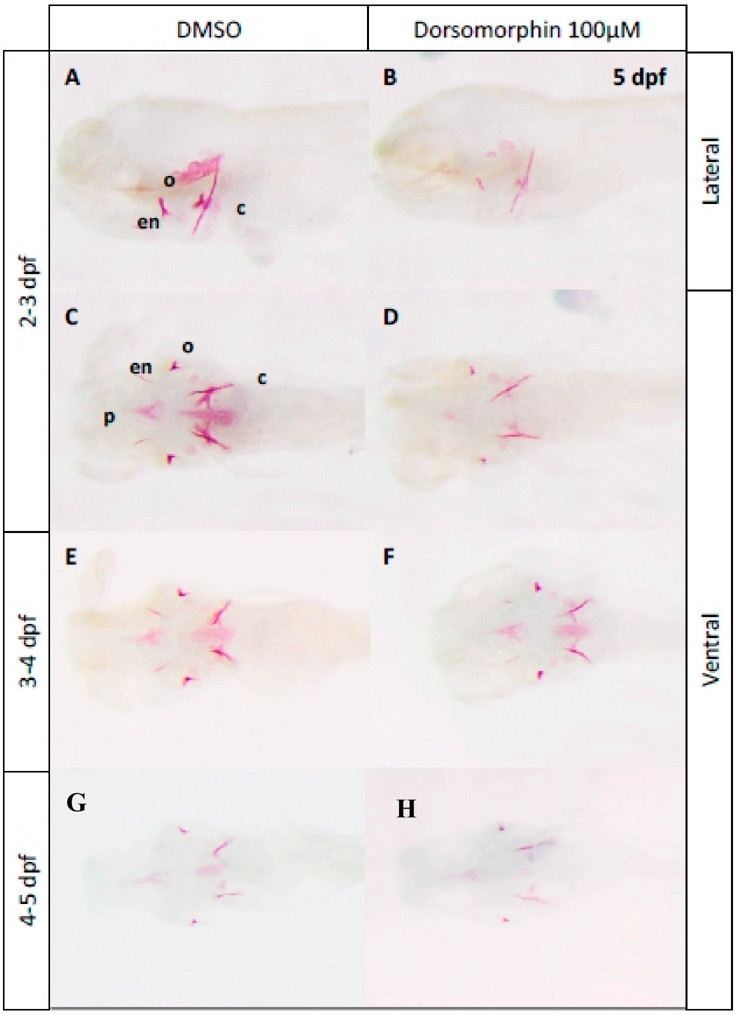

Figure S1. Duplicate experiment showing the effects of the BMP inhibitor dorsomorphin on bone mineralization. Alizarine red staining of 5 dpf larvae treated at 2, 3 or 4 dpf during 24 h with 100 µM dorsomorphin. Control embryos were treated with DMSO. Embryos treated at 2 dpf (**B** lateral view and **D** ventral view) show severe reduction of all mineralized bone pieces compared to the controls (**A** lateral view and **C** ventral view). The treatments starting at 3 dpf (**F** in ventral view) and 4 dpf (**H** in ventral view) lead to a reduction of bone mineralization, but to a lesser extend than at 2 dpf compared to the controls embryos (respectively **E** and **G** in ventral view). c: cleithrum; en: entopterygoid; o: operculum; p: parasphenoid. Scale bar: 200 µM.

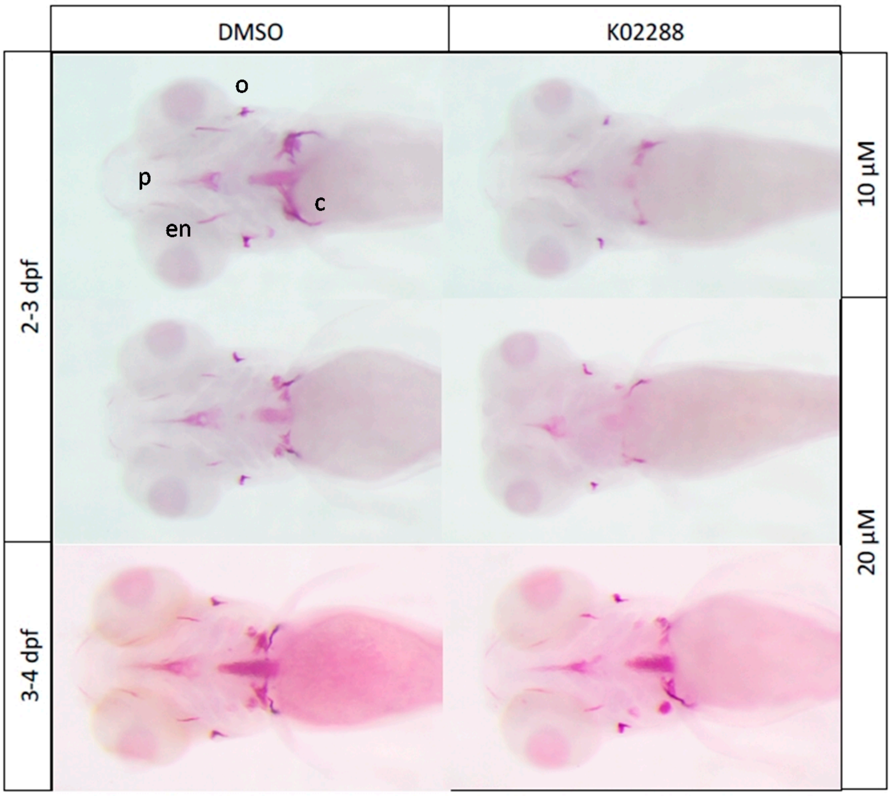

Figure S2. Duplicate experiment showing the effects of the specific BMP inhibitor K02288 on bone mineralization. Alizarine red staining of 5 dpf larvae previously treated at 2 or 3 dpf during 24 h with K02288 (10 and 20 µM). Control embryos were treated with DMSO. c: cleithrum; en: entopterygoid; o: operculum; p: parasphenoid. Scale bar: 200 µM.

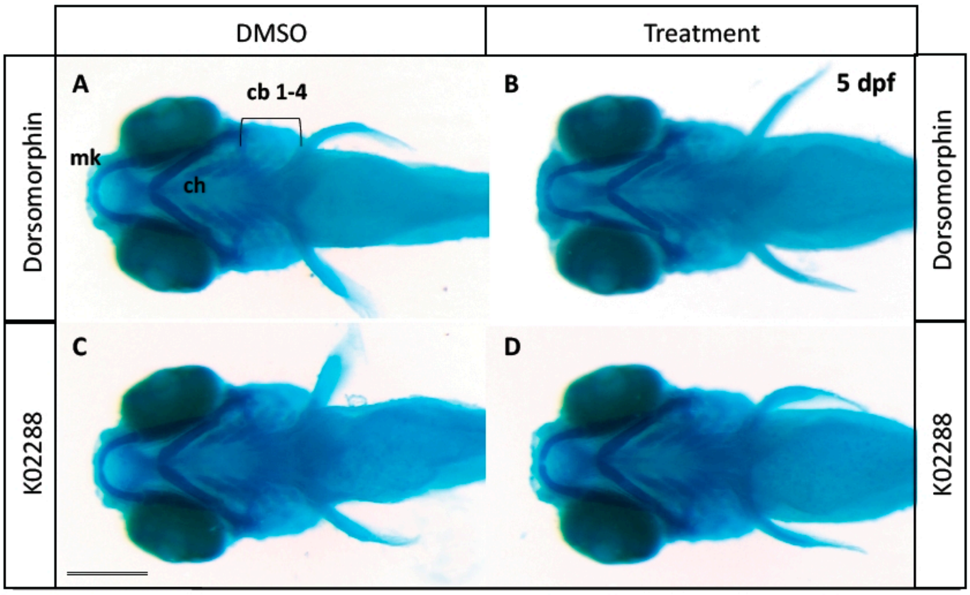

Figure 3. Duplicate experiment showing the effects of BMP inhibitor treatment at 2–3 dpf on cartilage formation. (**A**–**D**) Alcian blue staining of 5 dpf larvae previously treated at 2 dpf during 24 h with dorsomorphin (100 µM) or K02288 (20 µM). Control embryos were treated with DMSO.
